# The number and composition of work hours for attending physicians in Taiwan

**DOI:** 10.1038/s41598-020-71873-3

**Published:** 2020-09-10

**Authors:** Ray-E. Chang, Tsung-Hsien Yu, Chung-Liang Shih

**Affiliations:** 1 Institute of Health Policy and Management, College of Public Health, National Taiwan University, Taipei, Taiwan; 2 Department of Health Care Management, National Taipei University of Nursing and Science, Taipei, Taiwan; 3 Department of Medical Affairs, Ministry of Health and Welfare, Taipei, Taiwan; 4 Institute of Health Policy and Management, National Taiwan University, Room 639, No 17, Hsu-Chow Road, Taipei, 100 Taiwan

**Keywords:** Health policy, Health services

## Abstract

Long work hours among physicians is a worldwide issue in the healthcare arena. Previous studies have largely focused on the work hours of resident physicians rather than those of attending physicians. The purpose of this study was to investigate total work hours and the composition of those work hours for attending physicians across different hospital settings and across different medical specialties through a nationwide survey. This included examining differences in physician workload and its composition with respect to different hospital characteristics, and grouping medical specialties according to the work similarities. A cross-sectional self-reported nationwide survey was conducted from June to September of 2018, and the two questionnaires were distributed to all accredited hospitals in Taiwan. The number of physician work hours in different types of duty shifts were answered by medical specialty in each surveyed hospital. Each medical specialty in a hospital filled only one response for its attending physicians. The findings reveal that the average total work hours per week of an attending physician is around 69.1 h, but the total work hours and their composition of different duty shifts varied among hospital accreditation levels, geographic locations, emergency care responsibilities, and medical specialties. Because of the variance in the number and composition of attending physicians’ work hours, adjusting physician work hours to a reasonable level will be a major challenge for health authority and hospital managers.

## Introduction

Long work hours have been common across the globe and over many decades in the medical field^1^. This has even resulted in physicians going on strike^2,3^. As they can for anyone, long work hours over the long-term can cause burnout and health problems for physicians, including medical conditions (e.g., coronary heart disease and strokes), sleep disorders, depression, and substance abuse^4–7^. Even worse, recent studies have also shown that longer work hours not only harm physicians themselves, but also endanger a patient’s safety and life^8,9^. Organizations may also experience negative impacts such as low productivity, high rates of absenteeism, and increased use of sick leave, as well as physicians quitting their jobs, ultimately affecting job turnover significantly^10–12^. Understanding the work loads of a nation’s physicians would have important health policy implications.

One effective strategy for reducing the number of hours physicians work is to regulate their work hours, which is the strategy most widely adopted across the globe^13,14^. The harsh working conditions of resident physicians has received considerable public attention and resulted in subsequent reforms in the regulation of their working hours in Western countries since the 1990s^15,16^. Nevertheless, concerns about adequate training and debates over the outcome of patient care continues^17–19^. In Taiwan, the issue of long work hours and burnout for physicians has attracted public concern since 2011 because of several cases involving a physician’s sudden death. Following the global crusade to improve working conditions, Taiwan’s Ministry of Health and Welfare (MOHW) has implemented measures to restrict resident physicians’ work hours since August 2017, and resident physicians have been covered by the Labor Standard Act starting on September 1, 2019.

Attending physicians’ work hours, however, have not obtained as much attention as those of resident physicians, although their workload has been just as heavy. A systematic review synthesizing 122 studies found that the worldwide prevalence of physician burnout was 21.3%; quite a few studies even reported a burnout rate of more than 50%^20^. There is a high correlation between long work hours and physician burnout^21^. The majority of reported work hours of worldwide attending physicians has been around 50–60 h per week^22–26^, but some reported more than 70 h per week^27,28^. The duties of attending physicians are more diverse than those of residents, particularly in teaching hospitals: they have to supervise residents, interns, and medical students in addition to their clinical duty, and some also hold a faculty position in a medical school. In contrast, attending physicians in nonteaching community hospitals may only be responsible for clinical care and some administrative tasks. Moreover, healthcare needs vary substantially across regions. The size of the local population is a major factor in the volume of the healthcare demand, which results in different practicing arrangements for physicians. While urban physicians usually check a large number of patients within a short period of time, rural physicians see much fewer patients in the same amount of working hours. In addition, there are large variations in work hours among the different medical specialties^27^. On-call duty is an important factor contributing to the variation because the demand for urgent services differs substantially among different specialties (e.g., dermatology vs. obstetrics/gynecology). Thus, both the practice setting and nature of a physician’s specialty affect the work hours of attending physicians.

Consequently, it is necessary to understand both the length and composition of attending physicians’ work hours in order to give policy makers the information they need to create policies to relieve the burdensome workloads of physicians and to address the adequate supply of physicians. To the best of our knowledge, the definition of work hours for different duties, particularly in-house on-call, at-home on-call with or without return obligation, has been inconsistent or even unspecified in existing studies^29^. Most studies on physicians’ work hours have adopted a narrow definition of work hours, including the time for clinical service, research, administrative work, and other professional tasks^27,30–33^. A few studies adopted a broader definition and included things such as phone calls, emailing, and data-recording^34^. In addition, few studies had incorporated hospital characteristics in physician work hour surveys. Thus, the purpose of this study was to investigate total work hours and the composition of those work hours of attending physicians across different hospital settings and across different medical specialties through a nationwide survey.

## Results

Table 1 shows the distribution and responsiveness of respondent hospitals. A total of 152 hospitals participated in our survey: 17 (11.2%) of them were medical centers, 48 (31.6%) were regional hospitals, and 87 (57.2%) were community hospitals. Medical centers and regional hospitals had a higher participation rate (85.0% and 63.2%). Three-quarters of hospitals were located in urban areas, and for those hospitals the participation rate was around 34–37%. There were 19 (12.5%) hospitals with severe emergency care responsibility, 58 (38.2%) of them were with intermediate responsibility, 35 (23.0%) of them were with basic responsibility, and 40 (26.3%) were hospitals with no emergency responsibility. Our data also showed that the participation rate declined along with the level of emergency care responsibility.

**Table 1:** Distribution and representativeness of participating hospitals.

	All	Participated	Participation rate
Number of Hospitals	415	152	36.4
**Accreditation level**			
Medical center	20 (4.8)	17 (11.2)	85.0
Regional hospital	76 (18.3)	48 (31.6)	63.2
Community hospital	319 (76.9)	87 (57.2)	27.3
**Urbanity**			
Urban	304 (73.3)	114 (75.0)	37.5
Rural	111 (26.8)	38 (25.0)	34.2
**Emergency care responsibility**			
Advanced	38 (9.2)	19 (12.5)	50.0
Intermediate	75 (18.1)	58 (38.2)	77.3
Basic	75 (18.1)	35 (23.0)	46.7
None	227 (54.7)	40 (26.3)	17.6

Table 2 shows the distribution and responsiveness of respondent physicians. There were 2,365 valid replies collected. Of these, 691 (29.2%) were from medical centers; 1,026 (43.4%) were from regional hospitals; and the rest, 648 (27.4%), were from community hospitals. The results also revealed that 71.3% of respondents were from hospitals located in urban areas, and about 90% of them were from the hospitals with some level of emergency care responsibility. Regarding the medical specialty, while responses from emergency and laboratory departments accounted for 13%, responses from internal medicine and surgery departments were both in excess of 40%. The five specialties with the most respondents were general medicine (21.7%), general surgery (9.5%), pediatrics (5.7%), OB-GYN (4.9%), and orthopedics (4.8%). Also, participating hospitals were asked to assign an attending physician in each medical specialty who was the most representative of a physician workload to complete our work hour questionnaire, so that each questionnaire represented the current work hours and composition of work hours for each medical specialty. Therefore, our data represents the current work hours and work hour composition of 16,484 physicians, which accounted for 68.77% of physicians in Taiwan. From the perspective of hospital characteristics, the percentage of physicians represented in community hospitals and hospitals with no emergency care responsibility was comparatively low (< 40%), otherwise it was more than 50%. As for medical specialty, the percentage of physicians represented ranged from 60 to 76%. Therefore, our data are a fair reflection of the actual situation in Taiwan.

**Table 2:** Distribution and representativeness of responding physicians.

	All physicians in Taiwan	Number of returned questionnaires	Number of physicians represented	Percentage of physicians represented
Number of physicians	23,968	2,365 (100)	16,484	68.77
**Accreditation level**				
Medical center	8,605	691 (29.2)	7,617	88.5
Regional hospital	10,132	1,026 (43.4)	6,760	66.7
Community hospital	5,231	648 (27.4)	2,107	40.3
**Urbanity**				
Urban	20,571	1,932 (81.7)	14,665	71.3
Rural	3,397	433 (18.3)	1,819	53.6
**Emergency care responsibility**				
Advanced	12,448	725 (30.7)	9,533	76.6
Intermediate	6,924	1,146 (48.5)	4,919	71.0
Basic	2,000	267 (11.3)	1,000	50.0
None	2,596	227 (9.6)	1,032	39.8
**Specialty**				
Internal medicine department	11,833	1,093 (46.2)	8,188	69.2
Neurology	836	78 (3.3)	592	70.8
Pediatrics	1,285	134 (5.7)	885	68.9
General medicine	6,450	513 (21.7)	4,552	70.6
Psychiatry	770	92 (3.9)	568	73.8
Radiation oncology	271	40 (1.7)	197	72.7
Family medicine	1,371	102 (4.3)	838	61.1
Rehabilitation	619	106 (4.5)	389	62.8
Occupational Medicine	231	28 (1.2)	167	72.3
Surgery department	10,349	965 (40.8)	7,023	67.9
Neurosurgery	559	70 (3.0)	383	68.5
Plastic surgery	314	53 (2.2)	232	73.9
Orthopedics	1,188	113 (4.8)	743	62.5
OB-GYN	1,138	115 (4.9)	684	60.1
General surgery	4,046	224 (9.5)	2,804	69.3
Anesthesiology	978	94 (4.0)	679	69.4
Urology	742	90 (3.8)	484	65.2
Otolaryngology	522	72 (3.0)	375	71.8
Ophthalmology	579	79 (3.3)	435	75.1
Dermatology	283	55 (2.3)	204	72.1
Emergency medicine department	1,477	97 (4.1)	1,028	69.6
Emergency medicine	1,477	97 (4.1)	1,028	69.6
Laboratory department	734	210 (8.9)	545	74.3
Anatomical pathology	143	62 (2.6)	106	74.1
Radiology	274	92 (3.9)	201	73.4
Clinical pathology	152	11 (0.5)	112	73.7
Nuclear medicine	165	45 (1.9)	126	76.4

Table 3 shows the results of the length of each shift and total working hours per week and is stratified by hospital characteristics: accreditation level, urbanicity, and level of emergency care responsibility. The overall average total work hours for physicians per week was 69.1 h, and 74% of the total work hours, 51.2 h, were attributed to the scheduled working shift. In-house on-call shifts were the second largest fraction of total work, with 8.1 h spent on average, and accounted for 11.7% of total work hours. While 10.6% of the total work hours, 7.3 h, pertained to the at-home on-call service shift, only 3.5% of the total work hours, 2.4 h, were attributed to at-home standby shifts.

**Table 3:** Work hours composition: stratified by hospital accreditation level, urbanicity, and emergency care responsibility.

	Scheduled working		In-house on-call		At-home on-call service		At-home standby		Total work hours	
	Mean (SD)	p-value	Mean (SD)	p-value	Mean (SD)	p-value	Mean (SD)	p-value	Mean (SD)	p-value
Overall	51.2 (13.5)		8.1 (10.6)		7.3 (11.5)		2.4 (5.7)		69.1 (21.4)	
**Accreditation level**		< 0.0001^§^		< 0.0001^§^		< 0.0001^§^		< 0.0001^§^		< 0.0001^§^
(1) Medical center	56.8 (12.1)	(1) > (2) (1) > (3) (2) > (3)	6.4 (9.9)	(2) > (1) (3) > (1)	6.7 (9.8)	(2) > (1) (2) > (3)	3.4 (6.1)	(1) > (2) (1) > (3) (2) > (3)	73.2 (17.8)	(1) > (2) (2) > (3)
(2) Regional hospital	51.2 (11.6)		9.1 (10.8)		8.6 (13.0)		2.5 (6.2)		71.4 (20.9)	
(3) Community hospital	45.3 (15.0)		8.4 (10.8)		5.9 (10.3)		1.4 (4.1)		60.9 (23.6)	
**Urbanicity**		0.0025^¶^		< 0.0001^¶^		0.9088^¶^		0.0046^¶^		0.7216^¶^
Urban	51.6 (13.5)		7.5 (10.1)		7.3 (11.4)		2.6 (5.9)		69.0 (21.3)	
Rural	49.5 (13.2)		11.0 (12.0)		7.2 (11.6)		1.7 (4.4)		69.4 (21.8)	
**Emergency care responsibility**		< 0.0001^§^		< 0.0001^§^		< 0.0001^§^		< 0.0001^§^		< 0.0001^§^
(1) Advanced	56.4 (12.0)	(1) > (2) (1) > (3) (1) > (4) (2) > (3) (2) > (4) (3) > (4)	6.4 (10.0)	(2) > (1) (2) > (3)	6.9 (10.1)	(2) > (1) (2) > (3) (2) > (4) (1) > (4) (3) > (4)	3.4 (6.2)	(1) > (2) (1) > (3) (1) > (4) (2) > (3) (2) > (4)	73.1 (17.9)	(1) > (3) (1) > (4) (2) > (3) (2) > (4) (3) > (4)
(2) Intermediate	51.1 (11.8)		9.5 (10.8)		8.5 (12.7)		2.5 (6.1)		71.6 (21.0)	
(3) Basic	45.4 (14.4)		7.2 (9.9)		6.5 (11.0)		1.1 (3.2)		60.1 (22.8)	
(4) None	42.0 (16.7)		7.7 (11.2)		3.3 (7.8)		1.0 (2.5)		54.0 (22.5)	

^§^ANOVA, ^¶^t-test.

Tukey post-hoc comparison.

The length and composition of reported work hours varied significantly among different hospital accreditation levels. While physicians in medical centers had the longest average hours of scheduled working shifts and total work hours per week (56.8 and 73.2 h, respectively), physicians in community hospitals had the lowest number of hours of scheduled working shifts and total work hours per week (45.3 and 60.9, respectively). On the other hand, physicians in regional hospitals had the longest average hours of in-house on-call shifts (9.1 h) and at-home on-call service shifts per week (8.6 h). Regarding the hospital location, while physicians in urban hospitals and physicians in rural hospitals had almost identical total work hours, about 69 h, the composition of their total work hours was different in some of the shifts. Physicians in rural hospitals had significantly fewer scheduled working hours and at-home standby hours but more in-house on-call hours than their counterparts in urban hospitals. As for emergency care responsibility, physicians in hospitals with severe and intermediate emergency responsibility had significantly longer work hours than those in the standard and non-emergency care responsibility hospitals. The physician work hours on different duty shifts also varied significantly among hospitals with different levels of emergency care responsibility. While physicians in the severe level had the highest scheduled work hours and at-home standby hours, physicians in the intermediate level had the highest in-house on-call hours and at-home on-call service hours.

Table 4 reveals the total work hours and the composition of working hours among 23 medical specialties. A large variation in the length and composition of total work hours among different medical specialties was evident. The average total work hours per week varied between 51.1 to 84.5 h. The average hours of scheduled working shifts, in-house on-call shifts, at-home on-call service shifts, and at-home standby shifts varied from 45.8 to 56.1, 1.3 to 15.8, 1.1 to 18.3, and 0.4 to 5.3 h for different specialties, respectively. Examining the coefficient of variation, the number of hours of scheduled working shifts varied relatively less than other duty shifts. Since the absolute hours of at-home standby shifts were minute, the hours spent in this duty shift had a trivial effect on the total work hours. Therefore, the variations in the number of work hours of in-house on-call shifts and at-home on-call service shifts were the major factors causing the variations in the total work hours for attending physicians.

**Table 4:** The composition of working hours among clusters.

	Scheduled working		In-house on-call		At-home on-call service		At-home standby		Total
	Mean (SD)	%	Mean (SD)	%	Mean (SD)	%	Mean (SD)	%	Mean (SD)
**Cluster 1**	46.8 (1.0)	90.2	2.2 (0.8)	4.2	1.6 (0.6)	3.1	1.3 (0.4)	2.6	51.9 (0.8)
Rehabilitation	45.8 (14.8)	86.9	3.1 (7.5)	5.9	2.2 (6.7)	4.2	1.5 (4.8)	2.8	52.7 (19.9)
Occupational medicine	46.9 (12.9)	91.8	1.5 (3.8)	2.9	1.1 (2.6)	2.2	1.6 (5.0)	3.1	51.1 (16.1)
Dermatology	47.7 (11.5)	91.9	1.9 (4.0)	3.7	1.5 (4.3)	2.9	0.9 (2.5)	1.7	51.9 (15.6)
**Cluster 2**	52.1 (2.9)	85.9	2.8 (1.5)	4.6	4.3 (2.5)	6.9	1.6 (1.5)	2.6	60.8 (3.3)
Radiation oncology	51.9 (9.7)	86.1	1.8 (3.4)	3.0	5.3 (12.4)	8.8	1.3 (4.6)	2.2	60.3 (15.8)
Family medicine	48.7 (12.6)	83.4	5.9 (10.8)	10.1	3.4 (10.0)	5.8	0.4 (1.3)	0.7	58.4 (17.7)
Otolaryngology	53.1 (11.1)	81.3	4.7 (8.4)	7.2	5.2 (10.0)	8.0	2.3 (4.6)	3.5	65.3 (20.6)
Ophthalmology	48.8 (13.5)	84.0	2.3 (5.7)	4.0	4.4 (9.2)	7.6	2.6 (6.8)	4.5	58.1 (20.0)
Emergency medicine	55.7 (12.1)	93.9	1.9 (5.6)	3.2	1.1 (4.6)	1.9	0.7 (2.7)	1.2	59.3 (14.7)
Anatomical pathology	53.6 (10.4)	82.0	2.5 (5.0)	3.8	8.5 (12.6)	13.0	0.7 (2.4)	1.1	65.4 (17.3)
Radiology	48.9 (12.7)	76.4	3.3 (5.4)	5.2	7.0 (11.6)	10.9	4.9 (8.7)	7.7	64.0 (21.4)
Clinical pathology	56.2 (15.5)	92.7	1.3 (2.6)	2.1	2.5 (4.1)	4.1	0.6 (1.2)	1.0	60.6 (14.0)
Nuclear medicine	52.3 (8.6)	93.2	1.7 (5.0)	3.0	1.1 (5.1)	2.0	1.1 (4.9)	2.0	56.1 (13.4)
**Cluster 3**	50.1 (2.5)	70.7	11.0 (2.5)	15.5	7.8 (2.3)	10.9	2.1 (0.7)	2.9	71.0 (3.4)
Neurology	51.9 (11.2)	71.3	9.7 (9.2)	13.3	9.4 (12.2)	12.9	1.8 (4.4)	2.5	72.8 (16.4)
Pediatrics	53.1 (14.4)	74.2	11.1 (10.7)	15.5	4.7 (9.5)	6.6	2.7 (4.5)	3.8	71.6 (19.3)
General medicine	51.5 (13.2)	72.5	10.0 (10.6)	14.1	6.7 (10.1)	9.4	2.8 (6.9)	3.9	71.0 (21.3)
Psychiatry	46.4 (10.4)	71.8	10.6 (12.3)	16.4	6.0 (8.9)	9.3	1.6 (3.5)	2.5	64.6 (16.9)
Anesthesiology	48.6 (14.4)	65.2	15.8 (13.1)	21.2	9.1 (13.7)	12.2	1.0 (3.4)	1.3	74.5 (17.2)
Urology	49.3 (13.8)	69.1	8.8 (9.9)	12.3	10.7 (13.1)	15.0	2.5 (6.4)	3.5	71.3 (22.1)
**Cluster 4**	52.7 (2.1)	65.6	10.6 (0.9)	13.2	13.5 (3.3)	16.8	3.6 (1.1)	4.5	80.4 (2.3)
Neurosurgery	51.7 (12.0)	61.2	11.5 (11.1)	13.6	18.3 (14.9)	21.7	3.0 (5.0)	3.6	84.5 (18.9)
Plastic surgery	52.1 (12.4)	65.0	9.8 (11.9)	12.2	15.2 (14.5)	19.0	3.1 (6.8)	3.9	80.1 (17.0)
Orthopedics	54.7 (14.0)	68.8	9.6 (9.9)	12.1	11.3 (12.3)	14.2	3.9 (6.4)	4.9	79.5 (21.7)
OB-GYN	50.1 (16.8)	63.2	11.0 (12.9)	13.9	13.0 (14.5)	16.4	5.3 (7.4)	6.7	79.3 (24.2)
General surgery	54.9 (15.1)	69.8	11.3 (11.7)	14.4	9.9 (11.8)	12.6	2.7 (4.9)	3.4	78.7 (20.7)

Through the clustering process, the 23 medical specialties were sorted into four clusters. Cluster 1 included rehabilitation, occupational medicine, and dermatology, and the total work hours per week for physicians in these specialties was around 51–52 h. This cluster could be labeled as the specialties with relatively low scheduled working hours plus tiny amounts of work hours in other duty shifts. Cluster 2 included nine specialties: radiation oncology, family medicine, otolaryngology, ophthalmology, emergency medicine, anatomical pathology, radiology, clinical pathology and nuclear medicine. The total work hours of this cluster ranged from 56 to 65 h. Its specialties had a relatively higher level of scheduled working hours, and a moderate level of in-house on-call shifts and at-home on-call service shifts. Cluster 3 encompassed neurology, pediatrics, general medicine, psychiatry, anesthesiology, and urology. The total weekly work hours of this cluster ranged from 64 to 74 h. This group had longer scheduled working hours than cluster 1 and longer hours in three on-call duty shifts than clusters 1 and 2. The final cluster, cluster 4, included neurosurgery, plastic surgery, orthopedics, OB-GYN, and general surgery. The total weekly work hours of this cluster were the longest, ranging from 79 to 85 h. This cluster had the long work hours of scheduled working shifts and in-house on-call shifts, and also had the longest.

## Discussion

Understanding the composition of physicians’ work hours is the very first step to relieving physician burnout. As this survey is the first nationwide survey of attending physician work hours in Taiwan, the findings of this survey might help policy makers and hospital managers re-examine the reasonableness of physician work hours and re-think resource allocation. In summary, the findings of our study reveal that on average the total work hours of a physician is around 69.1 h per week, but that number varied among hospital accreditation level, urban/rural location, emergency care responsibility, and medical specialty. The composition of work hours also varied when viewed through the lens of hospital characteristics and medical specialties. In general, attending physicians at hospitals with higher accreditation levels, at hospitals with a higher level of emergency care responsibility, and in surgery-related specialties had longer work hour than others.

The tasks and responsibility undertaken by a hospital and the adequacy of its physician supply might be two major factors affecting the findings. Regarding hospital setting, the higher the accreditation level and the heavier the emergency care responsibility where a physician practiced, the longer the total hours a physician worked. This might be attributable to the fact that hospitals with a higher accreditation level or heavier emergency care responsibility had to assume more medical care duties and responsibilities, which resulted in heavier workloads for their physicians. Physicians in medical centers or hospitals with severe emergency care responsibility had to assume multiple duties including clinical, teaching, and research. These duties consume more time in scheduled working days. Cases in these hospitals might also be more complicated and urgent; therefore, physicians might be called back or consulted by phone more frequently than physicians in other types of hospitals. However, these hospitals also had a more generous supply of both residents and attending physicians. Thus, physicians practicing in these hospitals had higher scheduled working hours but not as many work hours of in-house on-call and at-home on-call service shifts as did physicians practicing in other types of hospitals. On the other hand, physicians practicing in regional hospitals or hospitals with intermediate emergency care responsibility had the highest work hours in in-house on-call and at-home on-call service shifts. This might be because the number of physicians working in these hospitals was not as sufficient as in medical centers or severe emergency care responsibility hospitals, but these hospitals still carried many medical care duties. Although community hospitals or hospitals with standard or none emergency care responsibility did not have a sufficient supply of physicians either, physicians practicing in these hospitals had high work hours in in-house on-call shifts but did not have as many work hours in at-home on-call service shifts as physicians in regional hospitals or hospitals with intermediate emergency care hospitals because less at-home on-call service duties were required. The reasons that the physicians in urban hospitals had more work hours in scheduled working and at-home standby shifts but fewer hours in in-house on-call shifts might be due to the aforementioned multiple duties, case complexity, and physician manpower supply.

The underlying factors of physician workload are probably largely related to the innate characteristics of a specialty, i.e., urgency of care, demand of the society directing or supporting the care process, and the market manpower supply. Services provided by specialties in cluster 1 are of relatively low urgency. Sufficient numbers of physician might also be a factor contributing to the low workload of physicians in this cluster. Thus, physicians in this cluster had relatively low work hours in all four duty shifts. On the other hand, services provided by specialties in cluster 4 involve a quite high level of urgency. Moreover, the large amount of care demand and a shortage of physicians may also be part of the cause for physicians’ long work hours in some specialties; for example, there is a large amount of care demand in orthopedics and general surgery and a shortage of physicians in OB-GYN and neurosurgery. Thus, physicians in this cluster had long work hours in all four duty shifts. As for cluster 3, the services in most of the specialties are also of a relatively high level of urgency of care. Physicians thus had long work hours in in-house on-call shifts and relatively long work hours in at-home on-call service shifts, but not as long as physicians in cluster 4. Cluster 2 encompasses two types of medical specialties: One type supports the care process rather than directing it, such as with clinical pathology, anatomical pathology, radiology, and nuclear medicine. The other type includes specialties such as ophthalmology, otolaryngology, and family medicine, which direct the care process, but the services might not be as urgent as clusters 3 and 4. Thus, for physicians in these specialties scheduled work hours made up a large portion of their workload but on-call or standby shifts were only a small portion. One unusual case was emergency medicine, which also had very little workload in on-call or standby shifts, because in Taiwan the rotating shift system is the most common scheduling one adopted by this specialty.

Are physicians’ work hours in Taiwan too long? In our survey, the average number of work hours per week was 69.1, which was longer than the 59.8 h reported in the turnover intention study of Tsai et al.^33^. A possible reason for lower total work hours in the Tsai et al. study could be the criteria for what they counted as work hours. Since no consensus on how to count work hours had been obtained in previous studies, the work hours of some duties might not have been included as work hours (e.g., at-home on-call service hours). Another study investigating 72 obstetricians who practiced in Taiwan’s hospitals and delivered more than 20 newborns per month found that the average weekly work hours were 80.14 h. Although the study was of a single specialty with a small sample size, the finding was similar to ours.

The number of total work hours in our study was also larger than in studies conducted in the US (which showed total work hours of around 50 h/week)^22–24^ and the UK (around 50 h/week)^25^, but was close to ones in Japan (around 60–70 h/week)^28,35,36^. The gaps might be due to the differences in healthcare systems that affect things such as physician employment (independent practice or in-house staff) and patient utilization regulations. Culture might also play an underlying role, since longer working hours were commonly observed in many job settings in Eastern countries. An international comparison of physician work hours among different healthcare systems would be interesting and warrants further research.

A reasonable number of work hours for physicians is critical for healthcare systems; fewer work hours can alleviate physicians’ fatigue and sleep deprivation, as well as burnout, and it might reduce medical errors and improve patient safety. However, it’s not a panacea. Literature has shown that some challenges might also come along with work-hour regulation, such as reducing the continuity of care, increasing the chance of cancellations of elective clinical activity, increasing intensity of the workload, and limiting flexibility in the management of working time^17,37,38^. Regarding the effect that reducing work hours has on the quality of patient care, a UK survey showed that 45% of respondents reported that reducing work hours has no effects on the quality of patient care, 40% reported a negative effect, and 15% a positive effect^17^. Thus, well-contemplated and comprehensive planning is imperative when conducting reforms to alleviate the physician workload.

Indeed, policy makers could contemplate several strategies, and these warrant further investigation. The first is to increase supply of physicians. To accomplish this, two approaches can be employed: increasing the enrollment of medical students and recruiting foreign physicians. Although this strategy is the most direct, it involves immense challenges. Recruiting foreign physicians will require addressing the compatibility and qualification of foreign medical training as well as the effectiveness of patient-physician communication. As for increasing medical student enrollment, it is extremely time-consuming, and increasing the number of qualified physicians may take 11–14 years to manifest.

The second strategy is to increase the number of workers who can take on some of a physician’s clinical tasks. Although nurse practitioners (NPs) have been allowed to conduct medical activities under a physician’s supervision in Taiwan since 2015, physicians still have a heavy workload. A limited NP supply might be partially attributable to a shortage of nurse manpower in the hospital industry. Thus, multiple sources of workers should be contemplated and examined. The profession of physician assistant/associate (PA), which prevails in many Western countries, such as the US, UK, Canada, Australia, and New Zealand, might be worth reviewing in Taiwan. The recent expansion of a PA workforce in those countries might provide some insights.

The third strategy is to consolidate regional physician resources. Since there is no gatekeeper in Taiwan’s National Health Insurance (NHI), patients are allowed to visit healthcare providers freely. This requires most hospitals to provide quite comprehensive and continuous outpatient and emergency services, which results in a heavy workload in many hospitals. For hospitals in rural areas or areas with an oversupply of physicians, physicians may have long work hours but treat a limited number of patients. A regional collaborating healthcare network might be a way to alleviate the workload of physicians in these areas. Hospital managers can also develop new scheduling approaches and reduce physicians’ non-clinical duties in their hospitals. Without question, reviewing the services currently provided and redesigning the healthcare service portfolio are also necessary for medical centers and regional hospitals under the NHI’s current policy of a 2% cut in outpatient services for 5 years.

The participation of only 152 hospitals, or 36% of hospitals in Taiwan, might raise concerns of a low response rate. However, these respondent hospitals were relatively large hospitals. The average number of staffed beds in the responding hospitals was 466, while the average number of staffed beds of non-responding hospitals was 192 (data not shown). Although the response rates of medical centers, regional hospitals, and community hospitals were 85%, 63%, and 27%, respectively, the attending physicians working in the responding specialty departments accounted for 88.5%, 66.7%, and 40.3% of attending physicians working in the medical centers, regional hospitals, and community hospitals. In total, the responses to our survey represent 16,484 physicians, accounting for 69% of hospital attending physicians in Taiwan. Further, according to the annual report of the National Health Insurance Administration, medical centers and regional hospitals each offered approximately 40% of all medical service (including all outpatient, inpatient, and emergency care services) in 2018. Therefore, although most of our responding physicians were from high-intensity hospitals, these hospitals also provide the vast majority of services in Taiwan. For that reason, we believe that the representativeness of the responses to our survey should be acceptable.

Nevertheless, some limitations exist in this study. Firstly, as with other cross-sectional study design, this study was only a snapshot of a process rather than a longer period of observation. Our results represented the situation at the time of investigation. An attempt to somewhat mitigate this concern was to widen the snapshot window by suggesting that physicians use a 3-month work schedule as a reference when filling out the work time questionnaire. Using a 3-month work schedule was also intended to reduce the risk of recall bias. Secondly, selection bias resulting from the work time questionnaire being filled by an appointed physician might be another concern. To reduce the risk of this bias, we suggested that the weekly work hours should be those of a typical attending physician working in that specialty department rather than based on the personal experience of the physician filling out the survey. In our design, the returned questionnaire was to be reviewed by the department chief in his/her specialty before it was returned to us. However, if the hospitals or department chiefs appointed inappropriate physicians to fill out the questionnaire, a bias might exist and affect the validity of this study. Finally, although the association with or impacts of work hours on physician health are important issues, these were not within the scope of this study. Future research is needed to investigate this subject in greater depth.

## Conclusion

The average number and composition of weekly work hours varied for physicians working in hospitals with different characteristics and in different medical specialties. In general, physicians working at hospitals with higher accreditation, working at hospitals with higher levels of emergency care responsibility, and working in surgery-related specialties had longer work hours than others. Physicians working in regional hospitals, hospitals located in rural areas, and hospitals with intermediate emergency care responsibility had a lower proportion of scheduled working shifts and a higher proportion of in-house on-call shifts to total work hours than others. Adjusting physician work hours to a reasonable level will be a major challenge in the future for health authorities and hospital managers.

Future studies can examine the effects of hospital characteristics and physician specialty on work hours of different working shifts. In addition, the number of hours worked by physicians might influence their mental and physical condition, and the relationship between these factors is worthy of further investigation.

## Materials and methods

### Study design

A cross-sectional self-reported survey supported by the Taiwan Ministry of Health and Welfare (Grant No: M06A7439) was conducted nationwide from June 15, 2018, to September 30, 2018, and distributed to all accredited hospitals. The target population of this study are the hospital attending physicians working in accredited hospitals in Taiwan. The survey inquired about the number of physician work hours and was answered by medical specialty in each surveyed hospital. Each medical specialty in a hospital filled in only one response for its attending physicians that was most representative of a physician workload in the most recent 3 months in that specialty department. The collected data were then analyzed to reveal the differences in the type of workload among physicians practicing in different hospital settings as well as in different medical specialties. Tests on differences in physician workload and composition with respect to different hospital settings were then conducted. Medical specialties were then clustered based on their work time similarities.

### Survey instrument

Hospitals with valid accreditation in 2018 were all invited to participate in our survey. Each invited hospital received two-part questionnaires, including two portions. The first part concerned basic information about the hospital, including hospital address, the number staffed beds, the number of attending physicians employed, the salary range of attending physicians, the number of specialties, the system for physicians’ leaves and paid vacation, and the physician practice insurance and job security system, as well as the average monthly outpatient and inpatient volumes, and was answered by the hospital administration department.

The second part concerned physician work hours and was answered by physicians. Each specialty department in a hospital was to appoint one physician to provide information on weekly work hours and the composition of the work hours (the definition of which is described below) based on the work schedule for the last 3 months of a typical and most representative attending physician working in that specialty department. The number of physicians in each specialty department was also requested. For those hospitals with teaching status, the specialties having resident physicians trained during the survey period were required to answer another copy of the same questionnaire for resident physicians. An appointed resident physician trained in that specialty was to be responsible for answering the resident physician’s copy. Since the issue of resident physicians is not within the scope of this study, the results of their work hours will be discussed in another study.

### Variables definition

This study was concerned with three types of important variables: work hours, hospital characteristics, and medical specialties, elaborated on below.

#### Work hours

In addition to regular scheduled work shifts, hospital attending physicians are assigned various types of on-call shifts. Although how to count work hours for the different kinds of shifts is not universally agreed to yet^39^, significant progress has been made through decades of efforts, and the crux of the issue is better understood. For the purposes of our study, we mainly referred to Germany’s Working Hours Act, and the European Union’s Working Time Directive (2003/88/EC) and its related documents^40–43^. We also reviewed judgments from the European Court of Justice (case C-303/98, C-151/02, C-14/04, and C-437/05)^44^ and a case of the California Court of Appeal, Second District^45^. Based on those sources we recognized four types of work shifts for hospital attending physicians in Taiwan: the regular scheduled working shift and three forms of on-call shifts. The on-call shifts were first categorized into in-house (or grounded to the duty site or assigned institution) shifts and at-home (no specified site) on-call shifts. We identified two forms of at-home on-call shifts, as they were essentially different in the obligation of returning to the duty site. In contrast to dated notions focusing on presence at the duty site or the intensity of work performed, the contemporary thought/ethos is more concerned with who controls the time, i.e., whether the employees are free to use the on-call time however they wish. When employees are prevented from using on-call time at home for their own purposes, the time is counted as working time.

#### Scheduled working shift

This includes all clinical, administrative, teaching, and research activities that attending physicians perform during their scheduled working hours (and any overtime that those duties might subsequently entail), such as providing patient care (both inpatient and outpatient); completing medical records; evaluating the performance of each member of the medical team; rounding with interns, residents, and students for bedside teaching; and reading or preparing research work at the duty site. Sometimes duties might be performed at other assigned institutions, such as class lectures at an affiliated university. All of the time spent on this shift counts as working hours.

#### In-house on-call shift

This is defined as those duty hours when attending physicians are off from their regular scheduled working shift but are required to be immediately available at their assigned institution. In general, physicians on this type of duty shift stay at the hospital in order to be able to act immediately should the need arise. Since physicians undertaking this duty shift have to remain on the employer premises and need to be attentive to calls, they cannot freely use the time as they wish. All of the time spent on this type of shift is regarded as working time^43,46^.

#### At-home on-call service shift

Physicians assuming this duty are outside the assigned institutions determined by their employers but are available to answer calls immediately or within a brief response time and obligated to return to the duty site within a certain amount of time. Thus, normally they would remain within a certain distance of the assigned institution. In addition, they are obliged to return to the duty site within a certain amount of time; thus, normally they remain within a certain distance of the assigned institution. Since the physicians are prevented from freely using this time at home however they wish, following the recent legal cases and official European Union documents, all of the hours of this type of on-call shift are considered working time^43^.

#### At-home standby shift

Physicians assuming this duty are also outside the assigned institutions determined by their employers but must be constantly reachable so that they can be called upon at short notice to act. Since the physicians are not obliged to return to the assigned institutions, they can manage their time without major constraints and are able to engage in personal activities and pursue their own interests. Therefore, only the time spent responding to calls or returning to hospitals and providing services, if the physicians choose to do so, is counted as working time.

#### Hospital characteristics

Hospital accreditation level, urbanization level, and the level of emergency care responsibility were used to describe the hospital characteristics. Hospitals in Taiwan are accredited as medical centers, regional hospitals, or community hospitals. The township each hospital is located in was recognized as either urban or rural. The emergency care responsibility of hospitals is sorted into four levels: advanced, intermediate, basic, and none. These characteristics are described in detail in Appendix 1.

#### Medical specialties

The twenty-three medical specialties officially certified by Taiwan MOHW were used as specialty classification gauge in this study. However, in many hospitals these were further divided into sub-specialties. Any response filled out by a sub-specialty was regarded as one response of the officially certified specialty it belonged to.

### Survey administration

For increasing the participation rate, the survey questionnaires were administered to all accredited hospitals with assistances from the Ministry of Health and Welfare. Moreover, in order to minimize the risk of the representativeness of physicians chosen for each specialty, and to ensure all respondents adopted the same definition of different types of work hours, ten orientations throughout Taiwan from May 15 to June 15 in 2018, and invited each accredited hospital to assign several physician heads and human resource director to attend one of these orientations. In addition to the detailed explanation on the definitions of four work shifts, we also explained how to choose a representative physician and verify the questionnaire filled by the representative physicians in the orientations. The representative physician should be selected in a formal meeting of his/her specialty, and the work hour questionnaire filled by the representative physician should also be agreed in another formal meeting of his/her specialty. Last, A telephone number and an account in an instant messaging application were also provided to answer questions or clarify any ambiguities that arose when respondents were filling out the questionnaires.

### Statistical analysis

All statistical analysis was performed by SAS 9.4. Frequency and percentage were used to describe the distributions of our responses. The means and standard deviations of physician work hours in different shifts and in totality were calculated. ANOVA tests were conducted to test the differences in total work hours with respect to various hospital characteristics. The hierarchical cluster analysis was conducted using centroid algorithm to group medical specialties through the similarities of the work hour composition. The number of clusters was decided by the researchers based on the Cubic Clustering Criterion (CCC) and rationality of the cluster numbers^47^.

### Ethical statement

The Institutional Review Board of the National Taiwan University Hospital approved the protocol that was used in the present study (protocol # 201911106W).

### Informed consent

Informed consent was acquired from all respondents, and all experiments were performed in accordance with relevant guidelines and regulations.

## Appendix 1

### Hospital accreditation levels

In Taiwan, hospitals provide outpatient, inpatient and emergency services. Hospital accreditation is conducted by Ministry of Health and Welfare (MOHW)^48^, and through passing a rigorous peer assessment process, a hospital can earn a 3-year-term accreditation. Three levels of accreditation can be accredited, i.e., medical center, regional hospital and community hospital. A medical center has to have 250 staffed beds or more and offer full-range clinical services provided by all twenty-three medical specialties officially certified by MOHW, both inpatient and outpatient. Moreover, a medical center has to bear the responsibility for tertiary care, in particularly, achieving the qualifications of the severe level of emergency care and cancer therapeutic quality care. Along with aforesaid requirements¸ medical centers are obligated to assume medical education role. Therefore, medical centers are affiliated with medical schools and has to earn the teaching hospital accreditation conducted by both Ministry of Education and Ministry of Health and Welfare.

In order to be accredited as a regional hospital, a hospital has to have 250 staffed beds or more and to assume tertiary care responsibility to the extent of achieving the qualification of intermediary level of emergency care. Since regional hospitals need not to provide full-range medical services, the size of regional hospitals is smaller than medical centers in terms of number of staffed beds. Although teaching role is not an obligation for regional hospitals, most regional hospitals assume the teaching duty and earn teaching hospital accreditation.

Community hospitals could be seen as an extended part of primary healthcare, their major mission is to provide clinical services to community. General internal, general surgery, obstetrics and gynecology, and pediatrics are the main services of community hospitals expected to delivery. Since no teaching role is expected, limited number of community hospitals earn teaching hospitals accreditation.

### Urbanicity

The township of each hospital located in was recognized as either urban or rural type according to the definition of urbanization published by Taiwan’s National Health Research Institutes. All 365 townships in Taiwan were classified into 7 clusters based on the following indicators: Population density (people/km^2^), proportion of people with a college or above degree, proportion of elder people over 65 years old, proportion of people who are agriculture workers, and the number of physicians per 100,000 people. Residential areas located in clusters of 1 to 3 were categorized as urban, and the others as rural.

### Emergency care responsibility

In Taiwan, a local health authority appoints the hospitals under its administration to assume different levels of emergency care responsibility according to a hospital’s emergency capability, local emergency care need, and the distribution of local emergency medical resources. The hospital emergency capability reviewed by a health authority is based on operating hours of emergency services provided, provision of specialties specified for different types of urgent cases, and response time for specified procedures in certain emergency cases. Hospitals with emergency care departments are assigned to assume one of three levels of emergency care responsibility: advanced, intermediate, and basic, and are required to earn the accreditation of hospital emergency care ability from the Ministry of Health and Welfare. Hospitals with advanced and intermediate emergency care responsibilities must offer 24-h emergency care services and have the ability to handle acute stroke, acute coronary heart disease, trauma, and high-risk pregnancy and newborns. Hospitals with advanced emergency care responsibility are required to have a comprehensive support team (General Surgery, General Medicine, Orthopedics, Pediatrics, Anesthesiology, Neurology, Neurosurgery, and OB-GYN) that can provide consultation within 60 min, and they are the last line of referral. Hospitals with intermediate emergency care responsibility are required to have General Surgery, General Medicine, and Orthopedics doctors that can provide consultation within 60 min. As for hospitals with basic emergency care responsibility, they have emergency departments only offering basic emergency services during scheduled hours. Therefore, the higher the level of emergency care responsibility a hospital assumes, the more sufficient the physician manpower required, the greater the number of required specialties, the better trained and skilled the support team, the more complicated the urgent cases being dealt with, and the shorter the required response time. Referring to the list of 2017 hospital emergency care accreditation, we categorized our participant hospitals into four levels: advanced, intermediate, basic, and none (those without any emergency care responsibility)^49^.
